# Editorial: Organ crosstalk in the pathophysiology and treatment of type-2 diabetes

**DOI:** 10.3389/fendo.2024.1379994

**Published:** 2024-02-22

**Authors:** Estela Lorza-Gil, Bilgen Ekim, Gencer Sancar

**Affiliations:** ^1^ Department of Internal Medicine IV, Division of Endocrinology, Diabetology and Nephrology, University Hospital Tübingen, Tübingen, Germany; ^2^ Institute for Diabetes Research and Metabolic Diseases (IDM) of the Helmholtz Center Munich at the University of Tübingen, Tübingen, Germany; ^3^ German Center for Diabetes Research (DZD e. V.), Neuherberg, Germany; ^4^ Joint Heidelberg-Institute for Diabetes and Cancer (IDC) Helmholtz Center Munich Translational Diabetes Program, Department of Internal Medicine, Heidelberg University Hospital, Heidelberg, Germany

**Keywords:** organ cross talk, diabetes, biomarkers for diagnosis and prognosis, extracellular vesicles (EVs), diabetic kidney disease, cardiomyokines, gestational diabetes

## Introduction

The interplay between organs and their signaling molecules, known as organ crosstalk, has gained significant attention in the context of regulation of metabolism and physiology. Accumulating evidence indicates that this crosstalk contributes not only to a healthy state but also to diseases such as type 2 diabetes (T2D) when not regulated properly. Understanding the function of the mediators in interorgan crosstalk could offer potential therapeutics and enable tracing disease progression by using these mediators as biomarkers.

In the realm of diabetes, diabetic kidney disease (DKD) stands out as a prevalent and consequential complication, affecting 20%-40% of individuals with diabetes. Given its substantial impact, there is a growing emphasis on identifying robust biomarkers for monitoring the progression of T2D and its associated complications. This pursuit aligns with the broader goal of personalized medicine, where tailored interventions can be planned by specific biomarker profiles. Extracellular vesicles (EVs) have emerged as promising candidates for biomarker discovery and therapeutic applications. Notably, EVs, particularly those carrying microRNA (miRNA) cargo, have been implicated in the modulation of pancreatic islet function and viability, holding potential as biomarkers for the diagnosis of Diabetes Mellitus. Moreover, EVs are recognized as key mediators of intra-islet crosstalk and interorgan communication, facilitating the maintenance of glucose homeostasis. Their involvement in the dynamic interplay between pancreatic islets and other organs, including the liver, adipose tissue, skeletal muscle, and placenta during pregnancy, further accentuates their significance in the broader landscape of metabolic health. Therefore, the multifaceted roles of EVs in both diagnostic and therapeutic domains underscore their potential as pivotal players in the pursuit of personalized medicine and comprehensive management of diabetic conditions.

## Cardiomyokines as modulators of metabolism

Cardiomyokines are a group of proteins secreted by cardiomyocytes, which play a significant role in modulating metabolism and maintaining metabolic homeostasis. One of the well-studied cardiomyokine atrial natriuretic peptide (ANP), a hormone secreted from the heart, plays a crucial role in regulating cardiovascular and renal functions, as well as exerting metabolic effects in adipose tissue, liver, and skeletal muscle. The study presented by Daniels et al. aimed to test the hypothesis that ANP reduces concentrations of the anorexigenic adipokine leptin in humans. In a controlled clinical trial, human ANP or matching placebo was intravenously infused into healthy men. The results showed that within 135 minutes of ANP infusion, there was a significant acute decrease in plasma leptin levels, along with a marked increase in free fatty acids, indicating activated lipolysis. Additionally, in human SGBS adipocytes, ANP suppresses leptin release. This study provides evidence that ANP reduces leptin levels in healthy humans, supporting its role as a cardiomyokine in a heart-adipose tissue axis. The findings offer valuable insights into the interaction between ANP and leptin in the human body, shedding light on their potential physiological and clinical implications.

## Biomarkers for diabetic kidney disease

T2D is merely a disease of elevated blood glucose levels and impaired insulin action. Patients with T2D commonly suffer from its late complications including retinopathy, liver fibrosis, neuropathy, cardiovascular disorders, and nephropathy. Acyl-coA-binding protein (ACBP) is a recently identified endocrine factor that regulates food intake and lipid metabolism (1). Circulating ACBP levels positively correlate with BMI, and age and also elevates under conditions of systemic inflammation such as sepsis (1–4). In this Research Topic, Schürfeld et al. identified ACBP also as an effector of kidney function. In patients with chronic kidney disease ACBP levels were elevated, which decreased upon hemodialysis treatment. Additionally, in patients that underwent unilateral nephrectomy, ACBP levels were increased; indicating that the kidney plays a critical role in determining the ACBP levels in the blood. Interestingly, in mouse models of kidney dysfunction such as db/db or eNOS-/-db/db mice, ACBP expression levels in the subcutaneous or visceral adipose tissue, kidney, liver, and hypothalamus remained unchanged indicating that the elevated ACBP levels upon kidney dysfunction are not due to its elevated expression but rather due to its elevated retention in the circulation.

In this Research Topic, serum C-peptide also emerged as a potential predictor of renal dysfunction in type 2 diabetes. Unlike Schürfeld et al., Sun et al. particularly focused on patients with type 2 diabetes who suffer from kidney failure employing a mathematical model approach. Sun et al. identified six predictive features by LASSO regression: HbA1c, hypertension, albumin-to-creatinine ratio, retinol-binding protein-to-creatinine ratio, quartiles of fasting C-peptide, and quartiles of fasting C-peptide to 2 h postprandial C-peptide ratio; followed by logistic regression algorithms to establish a statistically powerful prediction model for the occurrence of renal dysfunction in patients with T2D.

Overall, both ACBP and serum C-peptide claim to be potential biomarkers to monitor and predict kidney dysfunction in patients, respectively, which might pave the way to successful patient stratification methods in the near future and enable optimal treatment options in personalized medicine.

## Extracellular vesicles as crosstalk mediators in diabetes

Extracellular vesicles are diverse, membrane-bound particles that are released by cells into the extracellular space. They carry various biologically active molecules such as nucleic acids (DNA, miRNA, mRNA, tRNA), proteins, lipids, and metabolites, playing crucial roles in intercellular communication (5). In the last decade, EVs have emerged as a new category of biomarkers and promising therapeutic agents for the diagnosis and treatment of various diseases, including diabetes. This is attributed to their ability to modulate the function of beta cells and insulin action in peripheral insulin target tissues (6).

Wei et al. provided a comprehensive examination of how EVs mediate intracellular and interorgan crosstalk within pancreatic islets under both physiological and diabetic conditions. In the context of type 1 diabetes (T1D), characterized by immune cell infiltration, beta cell failure, and insulin deficiency, the authors gathered evidence indicating the involvement of EVs in the crosstalk between beta cells and immune cells. Pancreatic islets-derived EVs, containing autoantigens including GAD65, IA-2, insulin catabolites, and miRNAs can trigger inflammation and autoimmune responses, while EVs derived from immune cells, containing tRNA-derived fragments, can induce damage and dysfunction of beta cells. Regarding T2D, their review indicated that EVs derived from pancreatic islets can deliver bioactive materials including miR-29s, Mut-Reg1cp, and reduced miR-26a to peripheral tissues, initiating insulin resistance. Furthermore, EVs originating from peripheral tissues contribute to beta cell compensation and eventual failure. These data are complemented by the study of Wang et al., which proposed that altered miRNA in EVs released by the placenta contributes to the defective adaptation of beta cells during Gestational Diabetes Mellitus (GDM). In the study, exosomes derived from the placenta of GDM mice promoted beta cell apoptosis and impaired glucose tolerance in pregnant mice. The authors identified the EV cargo miR-320b as the potential contributor to beta cell dysfunction associated with GDM.

In summary, the studies covered in this Research Topic elucidated the intricate roles of EVs in diabetes pathogenesis, providing insights into the emerging applications of EVs in the maintenance of glucose homeostasis. Moreover, they provide concrete examples from human studies of how inter-organ crosstalk modulates metabolism and could be used as potential biomarkers.

## Author contributions

EL-G: Writing – original draft, Writing – review & editing. BE: Writing – original draft, Writing – review & editing. GS: Writing – original draft, Writing – review & editing.
